# Quality of web-based information at the beginning of a global pandemic: a cross-sectional infodemiology study investigating preventive measures and self care methods of the coronavirus disease 2019

**DOI:** 10.1186/s12889-021-11141-9

**Published:** 2021-06-14

**Authors:** Jenny Stern, Susanne Georgsson, Tommy Carlsson

**Affiliations:** 1 Department of Women’s and Children’s Health, Uppsala University, Uppsala University Hospital, SE-75185 Uppsala, Sweden; 2 Sophiahemmet University, Stockholm, Sweden; 3 The Swedish Red Cross University College, Huddinge, Sweden; 4 Department of Clinical Science, Intervention and Technology, Karolinska Institutet, Solna, Sweden

**Keywords:** Consumer health information, COVID-19, Primary prevention, Self care, Severe acute respiratory syndrome coronavirus 2, World wide web

## Abstract

**Background:**

reducing the spread and impact epidemics and pandemics requires that members of the general population change their behaviors according to the recommendations, restrictions and laws provided by leading authorities. When a new epidemic or pandemic emerges, people are faced with the challenge of sorting through a great volume of varied information. Therefore, the dissemination of high-quality web-based information is essential during this time period. The overarching aim was to investigate the quality of web-based information about preventive measures and self care methods at the beginning of the COVID-19 pandemic.

**Methods:**

in May 2020, consumer-oriented websites written in Swedish were identified via systematic searches in Google (*n* = 76). Websites were assessed with inductive content analysis, the JAMA benchmarks, the QUEST tool and the DISCERN instrument.

**Results:**

seven categories and 33 subcategories were identified concerning preventive measures (md = 6.0 subcategories), with few specifying a method for washing hands (*n* = 4), when to sanitize the hands (n = 4), and a method for sanitizing the hands (*n* = 1). Eight categories and 30 subcategories were identified concerning self care methods (md = 3.0 subcategories), with few referring to the national number for telephone-based counseling (*n* = 20) and an online symptom assessment tool (*n* = 16). Overall, the median total quality scores were low (JAMA = 0/4, QUEST =13/28, DISCERN = 29/80).

**Conclusions:**

at the beginning of the pandemic, substantial quality deficits of websites about COVID-19 may have counteracted the public recommendations for preventive measures. This illustrates a critical need for standardized and systematic routines on how to achieve dissemination of high-quality web-based information when new epidemics and pandemics emerge.

**Supplementary Information:**

The online version contains supplementary material available at 10.1186/s12889-021-11141-9.

## Background

The coronavirus disease 2019 (COVID-19) quickly escalated during the first quarter of 2020 as a significant threat to global public health, with a reported case fatality rate of > 4% and particularly high mortality rates among older persons and those with comorbidities [1]. To reduce the spread and impact of the pandemic, guidelines and studies recommend various preventive measures; i.e. interventions that members of the public are recommended to apply in their daily lives with the purpose to reduce the spread of the infection and stay healthy in order to lessen the personal impact of potential infection. There is a wide range of preventive measures that may be implemented for this purpose, and among the most common are thorough and frequent washing of hands, hand disinfection with sanitizers and distancing [2, 3]. As a response to the pandemic, countries around the world implemented to varying degrees different non-pharmacological preventive measures ranging from few and less drastic to several drastic and mandatory interventions [4]. Moreover, when an infection is suspected or confirmed, persons with mild infections are generally recommended to manage self care at home, such as isolating themselves and treat mild symptoms, e.g. cough, fever and breathlessness [5–7]. Achieving high compliance to both preventive measures and self care methods at the beginning of a new epidemic or pandemic requires sufficient cooperation and preventive actions in the daily lives of members in the general population. This calls attention to the importance of the dissemination of high-quality information developed to adequately inform and update the public [8].

Studies report a high level of public demand for web-based information about the prevention of communicable diseases causing epidemics and pandemics, such as COVID-19 [9, 10]. The Web has the potential to serve as a large and accessible platform for interactive, current and tailored health-related information [11]. However, using the Web for health-related information involves a widely acknowledged risk of encountering content of substandard quality [12, 13], potentially misleading information consumers or negatively impacting their ability to take actions in their daily lives. As a response to this risk, an increasing amount of researchers conduct observational studies assessing the quality of what is published on the Web [14–16]. One component in the field of supply-based infodemiology concerns utilizing a range of systematic methods with the purpose to evaluate the quality of online information [17]. Quality of web-based sources is a complex and multidimensional concept, involving a wide range of criteria used to evaluate online health-related information [18]. A large amount of studies assessing quality on the Web repeatedly report substantial quality deficits in regard to various medical topics [12], including infectious diseases [19, 20]. While much is still unknown about the quality of Web-based information about COVID-19, a limited number of studies have recently been published, all concluding that quality deficits are a current problems and that improvement are needed [21–25]. The pandemic is a significant global issue spanning across all continents, with considerable health-related consequences for the population regardless of geographical setting. So far, most published studies focus on websites written in the English [21–25] and Spanish [24, 25] language, raising questions about the quality of online information in other settings. The literature acknowledges a gap in research regarding evaluation of web-based information about health promotion, self-management and disease prevention [26]. Consequently, the overarching aim of this study was to investigate the quality of web-based information about the prevention and self care at the beginning of the COVID-19 pandemic, written in the Swedish language. Specifically, the following quality criteria were investigated: (i) comprehensiveness, (ii) transparency, (iii) quality of online sources about disease prevention, and (iv) reliability and quality of consumer health information about preventive measures and self care methods.

## Methods

### Design

This supply-based infodemiology study was cross-sectional and concerned information on websites about COVID-19 written in Swedish. Supply-based infodemiology concerns the quality of information distributed on the Internet with the purpose to inform members of the public about health-related topics [17]. The analysis was inspired by current recommendations for systematic analysis of consumer-oriented websites about health-related topics [26].

### Study context

The Public Health Agency of Sweden has a national responsibility for public health issues and to ensure good public health by disseminating scientifically based information to the public [27]. The first case of COVID-19 in Sweden was confirmed January, and up until March relatively few cases were confirmed. In March, the Public Health Agency of Sweden noted an increase in cases and issued the highest level of risk for spread of the disease in the Swedish society. Regular press conferences with information and updates were broadcasted for the public to access. The agency provided public recommendations how to reduce spread of the infection and flatten the curve of new confirmed cases in order to minimize number of patients in need hospitalization. Mainly, recommendations concerned thorough and frequent washing hands with soap and water, distancing of at least two meters from others, refraining from social activities with greater numbers of participants, avoiding unnecessary travel, working remotely from home, and isolating at home/avoiding social contacts when presenting with symptoms or when > 69 years of age. The public is referred to a national telephone line (telephone number 113 13) for information about COVID-19 for questions not related to symptoms and another national telephone line (telephone number 1177) if they or a relative is unwell and in need of healthcare consultation with a registered nurse. In case of an emergency or danger to life, the public is referred to the national emergency number (telephone number 112). There is a very high Internet accessibility in Sweden and a large majority of Swedes use the Web to find information, including about health-related topics [28].

### Data collection

Websites about COVID-19 were identified via Google, the most popular search engine in Sweden with approximately 97% of the Swedish population reporting they use it to search for web-based information [28]. The searches were designed to replicate search patterns observed in the general population: using multiple varieties of search strings, screening the first links in the hit list before performing a new search in the search engine, and procuring the information presented in the first web page of each link in the hit list without moving on to other links found in the web pages accessed via the hit list [29–32]. In total, 17 search strings were chosen that were considered to represent searches used in the general population (Additional file 1). These search strings were designed by the last author and were inspired by searches presented in Google Trends, by exploring popular rising and top Swedish as well as global search terms related to COVID-19. No quotation marks or other search engine operators were used during the searches.

The first twenty hits in the hit list presented in the search engine were screened for inclusion, meaning that 340 hits were screened in total. All searches were performed in May 2020 using the Web browser Google Chrome, set to incognito mode in order to limit the impact of previous searches on the computer. Inclusion criteria were the following: (1) contain text-based information about the prevention and self care of COVID-19, (2) written in the Swedish language, (3) provide information aimed toward the general population, and (4) accessible without any password or payment requirements. Social media content, blog posts, discussion boards, and other websites containing text-based material written by laypersons to communicate with peers and share experiences were excluded, because these were not considered to have the purpose of disseminating information about preventive measures and self care methods to the general population. For the same reason, websites written for health professionals were also excluded. Website domain was not given consideration when screening for inclusion.

**Fig. 1:**
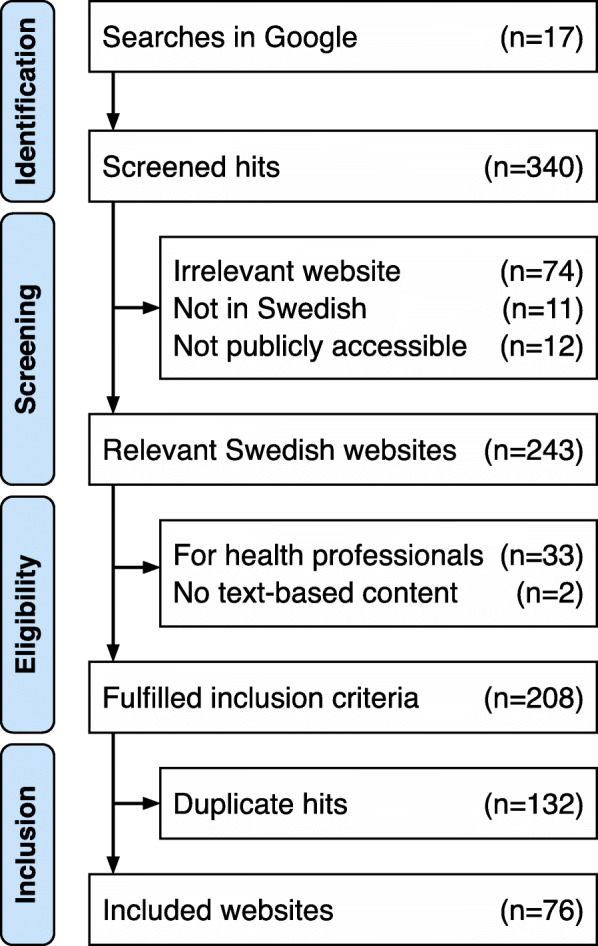
Identification and inclusion process for Swedish websites about COVID-19

Of the 340 hits screened for inclusion, 97 hits were excluded because they were irrelevant (i.e. did not contain any information related to COVID-19), not publicly accessible, or not written in Swedish. Of the remaining 243 relevant hits, 35 were excluded because they were written for health professionals or did not contain any text-based content. After correcting for duplicate hits (*n* = 132), 76 websites were included in the final sample (Fig. 1). In order to save the content of each website at the time of data collection, all were captured with NCapture in May 2020.

### Data analysis

The following quality criteria were assessed: (i) comprehensiveness, (ii) transparency, (iii) quality of online sources about disease prevention, and (iv) reliability and quality of consumer health information about preventive measures and self care methods. Additional File 2 presents details concerning the instruments used for quality assessment. Data were analyzed with descriptive statistics and the Kruskal-Wallis test (Dunn’s test with the bonferroni correction as post hoc analysis) was used to explore differences between website affiliation (i.e. the type of organization or company that is hosting the website). Associations between investigated quality variables were calculated with Pearson’s correlation coefficient. *P* < .05 was considered statistically significant. Statistics were calculated with RStudio (version 1.0.143).

#### Comprehensiveness (inductive content analysis)

Comprehensiveness concerns the range of topics covered by a website [18], which was analyzed with manifest content analysis [33]. An inductive approach was applied because we did not want to be constrained or influenced by any preconceived theories or models. The analysis included the following steps: (1) each website was read carefully to gain an overall understanding about the content, (2) meaning units were identified, defined as words, sentences or paragraphs about a certain topic, (3) all meaning units were considered highly manifest in nature and were therefore directly placed into externally heterogeneous categories and sub-categories, illustrating collections of meaning units with an internally homogeneous content. The categorization was managed with Nvivo (version 12). The number of identified categories and subcategories were counted for each of the included websites as an indication of the range of different preventive measures or self care methods mentioned in the websites.

#### Transparency (JAMA benchmarks)

Transparency concerns the disclosure of details about the production of the information that may influence the ability to make informed choices [18], which we assessed with the Journal of the American Medical Association (JAMA) benchmarks. The instrument assesses four basic quality criteria illustrated in benchmarks: authorship, attribution, disclosure and currency [34]. The number of adhered benchmarks were summarized for each website, resulting in a total score of 0 to 4 adhered benchmarks.

#### Quality of online sources about disease prevention (QUEST)

The QUality Evaluation Scoring Tool (QUEST) was used to assess quality of online sources about disease prevention, based on a set of six different indicators: authorship, attribution, conflict of interest, currency (i.e. timeliness), complementarity and tone. QUEST is a reliable and valid instrument, suitable for assessment of health-related online content about a variety of topics including disease prevention. The six quality indicators are assessed through seven questions, rated on a scale in which higher scores represent higher quality. Each question in the tool is weighted according to how critical it is to the overall quality and ethical implications, generating a total score between 0 and 28 [35].

#### Reliability and quality of consumer health information (DISCERN)

The DISCERN instrument, a reliable and valid tool [36, 37] extensively used in the literature to assess quality of online consumer health information [36], was used to systematically analyze reliability and quality of information about preventive measures and self care methods. The instrument involves the subscales reliability (eight questions including one optional), information about health-related options (seven questions), and overall quality (one question). Reliability concerns aspects assessed to judge if the publication can be trusted as a source of information, while quality of information focuses on specific details about the covered topics [38]. In its original format, the instrument is intended to assess quality of information about treatment options. Thus, the wording of the questions in the second subscale was somewhat modified so that the questions concerned preventive measures and self care methods, please see Additional File 2 for details. Each question in the instrument is rated on a scale from 1 (no/low quality) to 5 (yes/high quality), generating a total score between 15 and 80. Higher scores represent higher quality.

The literature suggests that the DISCERN instrument can be used to assess information regardless of the background and qualifications of the assessor [39]. All but one of the questions in the instrument concerns the aspects mentioned in the specific publication [38], meaning that the websites were assessed in regard to the preventive measures/self care methods described therein, and that the questions were rated based on the website as a source of information about both preventive measures and self care methods.

#### Assessment procedure

The last author was responsible for the assessments of all websites in regard all of the investigated quality criteria. The first author performed a separate assessment of > 20% (first 16 of the included websites) websites in regard to JAMA, QUEST and DISCERN. Interrater reliability of the assessments of the subset was calculated with intra-class correlation. According to the literature, interrater reliability ranges from < 0.4 (poor agreement), 0.4–0.59 (fair agreement), 0.6–0.74 (good agreement), to 0.75–1.0 (excellent agreement) [40]. Based on these conditions, the interrater reliability between the two assessors in this study was determined as excellent, with 0.85 for the total number of adhered JAMA benchmarks, 0.98 for the total DISCERN score and 0.85 for the total QUEST score. Based on the high interrater reliability, the last author continued to single-handedly analyze the remaining websites. Please see the section ‘Author’s information’ for details concerning the qualifications and backgrounds of the assessors who evaluated the websites (first and last authors).

## Results

### Website affiliation

The included websites were affiliated with the government (*n* = 19), health care services (*n* = 17), newspapers (*n* = 17), information websites, i.e. produced by independent companies with the sole purpose to provide web-based information (*n* = 9), pharmacies (*n* = 5), and nine websites were categorized as having other affiliation (humanitarian organizations, *n* = 2; universities, n = 2; insurance company, *n* = 1; medical products company, n = 1; online health food store, n = 1; patient organization, n = 1; wiki page, n = 1).

### Comprehensiveness (inductive content analysis)

#### Preventive measures

**Fig. 2:**
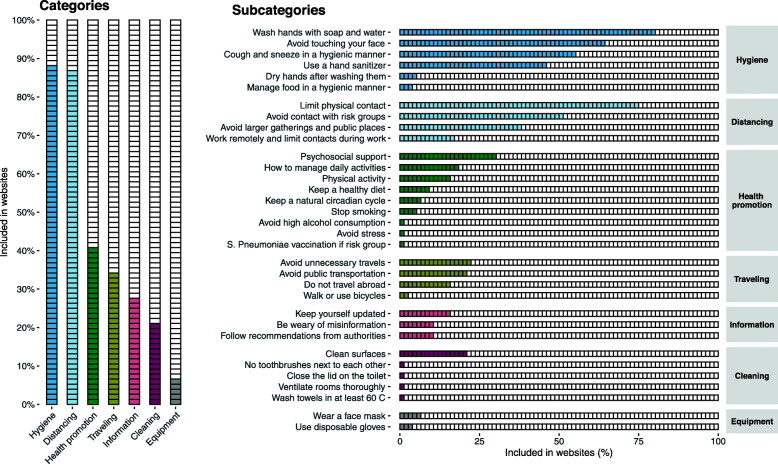
Prevalence of categories and subcategories about preventive measures in the included websites (n = 76)

Seven categories and 33 subcategories were identified about preventive measures (Fig. 2), and the median number of included subcategories about preventive measures was 6.0 (Table 1). The most prevalent categories were personal hygienic measures (*n* = 67, 88%) and physical distancing (*n* = 66, 87%), while the least prevalent were household cleaning (*n* = 16, 21%) and protective equipment (*n* = 5, 7%). There were significant differences in number of included subcategories between affiliations (*X*^2^ = 12.6, *P* = .03), with websites affiliated with the government (*P* < .01) including more subcategories (Md = 9.0) compared to news websites (Md = 4.0), Additional File 3. In regard to specified directions (Table 2), more than half described when to wash hands (*n* = 52, 68%) and that you should sneeze into the elbow (*n* = 39, 51%), but few specified a method for washing hands (*n* = 4, 5%), when to sanitize hands (n = 4, 5%), and a method for sanitizing hands (*n* = 1, 1%).

**Table 1:** Investigated quality variables for the websites (*n* = 76) [minimum to maximum achievable score in square brackets]

Quality variable/instrument	Md (IQR)	Range
Comprehensiveness		
Included subcategories about preventive measures [0–33]	6.0 (5.0)	0–16
Included subcategories about self care [0–30]	3.0 (3.25)	0–17
Total number of included subcategories [0–63]	10.0 (8.0)	1–26
DISCERN		
Subscale 1 (reliability) [7-40]	14.5 (5.0)	9–30
Subscale 2 (information about prevention and self care) [7-35]	12.0 (5.0)	7–21
Subscale 3 (overall quality) [1-5]	2.0 (1.0)	1–4
Total score [15–80]	29.0 (9.0)	19–53
JAMA benchmarks		
Number of adhered benchmarks [0–4]	0 (1.0)	0–4
QUEST		
Authorship [0–2]	0 (1.0)	0–2
Attribution [0–9]	3.0 (3.0)	0–9
Attribution 2^1^ [0–2]	1 (−)	1
Conflict of interest [0–6]	6.0 (0)	0–6
Currency [0–2]	2.0 (2.0)	0–2
Complementarity [0–1]	1.0 (1.0)	0–1
Tone [0–6]	3.0 (0)	0–6
Total score [0–28]	13.0 (3.25)	6–23

^1^Follow-up attribution score only applicable for two websites

**Table 2:** Specified directions about hygienic measures described in the websites (*n* = 76)

Sub-category	Specified directions		n (%)
Wash hands with soap and water	When to wash hands		52 (68)
		Often, regularly^1^	46 (61)
		Before and after meals	17 (22)
		After being outdoors and in high-risk areas	16 (21)
		After bathroom visits	14 (18)
		After sneezing or coughing	7 (9)
		After touching the same surfaces as a sick person	1 (1)
		After travelling	1 (1)
		Before and after breastfeeding	1 (1)
		Before touching your face	1 (1)
		After doing the laundry	1 (1)
		When hands are visibly dirty	1 (1)
	How long to wash hands		28 (37)
		At least 20 s	24 (32)
		At least 30 s	5 (7)
	Method for effectively washing hands		4 (5)
		All sides of the hands and the fingers	4 (5)
		Under jewelry	2 (3)
		Under the nails	1 (1)
Cough and sneeze in a hygienic manner	Cough and sneeze into your elbow		39 (51)
	Cough and sneeze into a tissue		28 (37)
Use a hand sanitizer	What type of sanitizer to use		6 (8)
		Solution with at least 60% alcohol	5 (7)
		Solution with hydrogen peroxide	1 (1)
	When to sanitize hands with disinfectant		4 (5)
		Often, regularly^1^	3 (4)
		After doing laundry	1 (1)
	Method for effectively sanitizing hands		1 (1)
		Rub between hands until they are dry	1 (1)

^1^Not specified how often

#### Self care methods

**Fig. 3:**
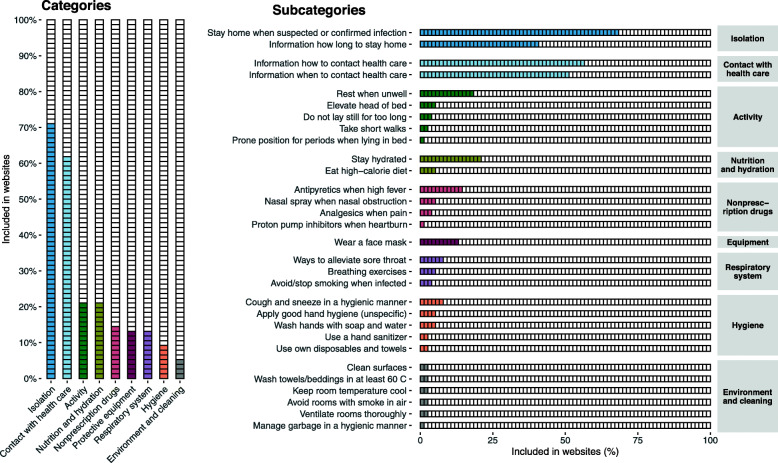
Prevalence of categories and subcategories about self care methods in the included websites (*n* = 76)

Eight categories and 30 subcategories were identified about self care (Fig. 3), and the median number of included subcategories about self care methods was 3.0 (Table 1). The most prevalent categories were isolation when presenting symptoms (*n* = 54, 71%) and contact with health care (*n* = 47, 62%), while the least prevalent were information about hygiene (*n* = 7, 9%) and environment and cleaning (n = 4, 5%). No significant differences were found in regard to the number of subcategories about self care methods between website affiliations (Additional File 3). There was high variability in regard to specified directions about isolation and contact with health care services, as only one of the specified directions was mentioned by > 50% of the websites (Table 3). A small proportion referred to the national number for telephone-based health care counseling (*n* = 20, 26%) and online tools for self-reported symptom assessment (*n* = 16, 21%). In total, 20 (26%) prompted readers to contact health care services if feeling critically ill and fewer specifically referred to the national emergency telephone number (*n* = 13, 17%).

**Table 3:** Directions about isolation and health care contact when presenting symptoms, described in the websites (n = 76)

Category	Sub-category	Specified directions	n (%)
Isolation when presenting symptoms	Stay home when suspected infection	All persons with symptoms	40 (53)
		If feeling sick	31 (41)
		Important when working in health care	5 (7)
		Keep distance when sick	3 (4)
		When suspecting having contracted the infection	3 (4)
		Do not have guests over when sick	2 (3)
		If you get ill after staying in a high-risk area	1 (1)
		When diagnosed with COVID-19	1 (1)
	How long to stay home when suspected or confirmed infection	Two days after end of symptoms	28 (37)
		During illness	5 (7)
		Can return to work after 2 or 7 days if certain symptoms persist^1^	5 (7)
		At least for 14 days when confirmed diagnosis	4 (5)
		At least for 7 days after onset of first symptom	3 (4)
		Stay home for a few days after end of symptoms	1 (1)
		Stay home for one day after end of symptoms	1 (1)
Contact with health care	Information how to contact health care	Through the national number for telephone-based health care	20 (26)
		Reference to self-reported online tool for symptom assessment	16 (21)
		Call the national emergency telephone number when critically ill	13 (17)
		Turn to online health care services for medical care	11 (14)
		Reference to call primary health care centre	10 (13)
		Seek care at the emergency room if health care centre is closed	4 (5)
	Information when to contact health care	Limit contact with health care when having non-serious symptoms	29 (38)
		Contact health care services if feeling critically ill	20 (26)

^1^Dry cough, loss of taste, loss of smell

### Transparency (JAMA benchmarks)

**Fig. 4:**
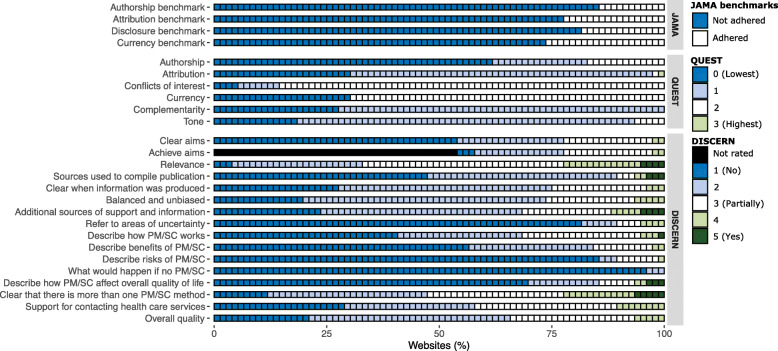
Distributions of quality scores for the websites (n = 76). *PM/SC: preventive measures/self care; QUEST items not weighted*

The median number of achieved JAMA benchmarks was 0 (Table 1), indicating insufficient quality. Authorship was not mentioned in 56 (74%), while information in other websites were authored by journalists (*n* = 10, 13%), editors (*n* = 7, 9%), PhD in medical sciences (n = 2, 3%) and physicians (n = 1, 1%). In total, 67 (88%) did not describe any review process, while information in other websites were reviewed by physicians (n = 7, 9%), medical editor (n = 1, 1%) and editor (n = 1, 1%). There were significant differences in regard to number of achieved JAMA benchmarks between affiliations (*X*^2^ = 32.3, *P* < .01). Information websites (Md = 3.0) had significantly more achieved benchmarks compared to websites affiliated with the government (Md = 0, *P* < .01), health care services (Md = 0, *P* < .01), pharmacies (Md = 0, *P* < .01) and other websites (M = 0, *P* < .01). Websites affiliated with newspapers (Md = 1.0) had significantly more achieved benchmarks compared to websites affiliated with the government (Md = 0, *P* < .01), health care services (Md = 0, *P* = .01) and pharmacies (Md = 0, *P* = .02), Additional File 3. Across all four benchmarks, less than 30% of the included websites adhered to the criteria (Fig. 4).

### Quality of online sources about disease prevention (QUEST)

The median total QUEST score was 13.0 (Table 1). Particularly low median scores were found for authorship (0 out of 2.0), attribution (3.0 out of 9.0), and tone (3.0 out of 6.0). There were significant differences in regard to total QUEST score between website affiliations (*X*^2^ = 15.7, *P* < .01). Information websites (Md = 16.0) had significantly higher total QUEST score compared to websites affiliated with the government (Md = 12.0, *P* = .04), health care services (Md = 12.0, *P* = .02) and pharmacies (M = 9.0, *P* < .01), please see Additional File 3 for detailed information. Most websites had a total QUEST score < 17 (Fig. 2). The websites with the highest total QUEST score also had moderate to high total DISCERN scores and were affiliated with newspapers (*n* = 16 and 14 included subcategories, respectively). In total, > 50% of the included websites were rated lowest possible score for authorship, > 25% were rated lowest possible score for attribution, currency and complementarity, and 86% were rated highest possible score for conflicts of interest (Fig. 4).

### Reliability and quality of consumer health information (DISCERN)

**Fig. 5:**
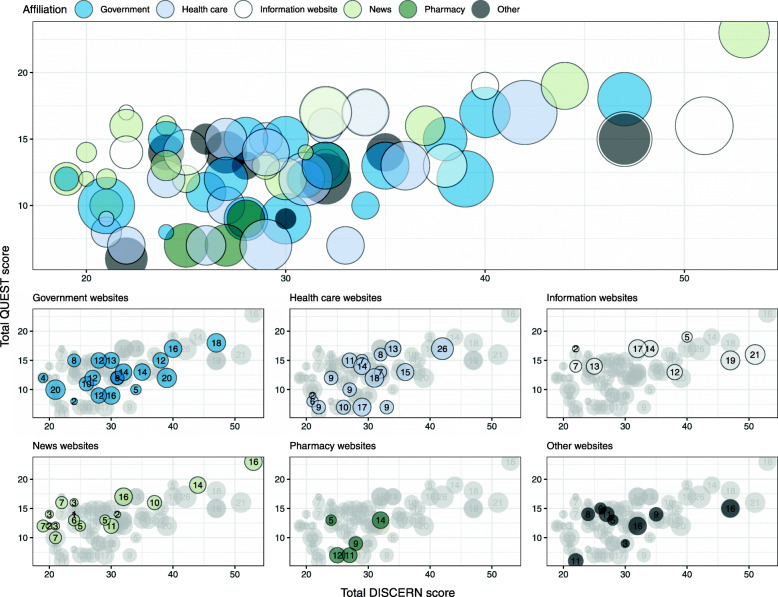
Bubble plot of total QUEST and DISCERN scores with number of included subcategories. *Size of and numbers in bubbles indicate the number of included subcategories, illustrating the range of preventive measures and self care methods mentioned in each website. Colors of bubbles indicate website affiliation*

The median total DISCERN score was 29.0 out of a maximum achievable score of 80, indicating low overall quality (Table 1). There were no significant differences in regard to subscale 1 (reliability), subscale 2 (quality of information), subscale 3 (overall quality) or total DISCERN score between website affiliations (Additional File 3). Most websites had a total DISCERN score < 40, and websites with particularly low DISCERN scores had low total QUEST scores and included few subcategories of comprehensiveness (Fig. 5). The websites with the highest total DISCERN scores also had high QUEST scores and were affiliated with a newspaper (*n* = 16 included subcategories) and an information website (*n* = 21 included subcategories). There was a significant moderate correlation between total DISCERN score and number of included subcategories (r = 0.61, *P* < .01).

In total, > 50% of websites were rated the lowest possible scores for 13 of 16 DISCERN questions, i.e. a score of 1 or 2 (Fig. 4). In regard to overall quality (subscale 3), 50 (66%) websites had low quality (a score of 1 or 2), 21 (28%) had moderate quality (a score of 3), and 5 (7%) had high quality (a score of 4). Two questions had > 50% of websites with a score of > 3, indicating at least moderate quality (*if the publication is relevant* and *if it is clear that there is more than one preventive measure/self care method*)*.*

## Discussion

The aim was to investigate the quality of Swedish web-based information about preventive measures and self care methods at the beginning of the COVID-19 pandemic, identified through searches in the widely popular search engine Google. Five sets of quality variables were investigated using inductive content analysis and three instruments for systematic quality assessment. A high variability was observed in regard to comprehensiveness and few websites included specific directions. Low quality was seen across all three instruments and significant differences between website affiliations were identified in two of the instruments, with information website affiliation generally having higher quality and government or health care affiliation not having higher quality than other websites.

The global COVID-19 pandemic required considerable preventive measures implemented in the daily lives of all members in the population [41]. This involved a significant challenge and responsibility on members of the general population at the beginning of the pandemic, who needed to sort through a great volume of information during a new and unfamiliar situation. Understandably, the public had a substantial demand for reliable and comprehensive information regarding how they needed to change their behavior to reduce the risk of contracting and spreading the infection [9, 10, 42]. Access to the Web is generally high across the world and many use it to find health-related information [28, 43, 44], indicating a potential to use the Web as a platform to effectively disseminate high-quality information intended for the public. The increased accessibility of the Web across socio-demographic populations has shifted focus from what has been referred to as a digital divide towards the skills needed to identify and evaluate web-based information [45]. When desiring health-related information on the Web, users need to be able to confidently search for and identify trustworthy sources. However, previous studies have revealed that lay persons employ ineffective and problematic search strategies when asked to find health-related information on the Web [29–32]. In this study, we excluded almost 40% of the hits when using search patterns similar to the public because they were irrelevant, written in other languages or written for health professionals. This indicates that persons who search for web-based information about COVID-19 may experience difficulties identifying relevant information developed for the public. Giving further weight to this hypothesis, many members of the public report that they experience difficulties judging the trustworthiness of sources about COVID-19 [46]. It is probable that searching for public information about COVID-19 is difficult and future studies should explore this among end-users in the general population.

The quality deficits observed in this study echoes what has been reported regarding websites about COVID-19 written in the English [21–25] and Spanish [24, 25] language. Quality of websites is a multidimensional concept involving various aspects that can be assessed through different methods. Four studies assessing websites with the DISCERN instrument [21–24] and two studies assessing websites with JAMA benchmarks [21, 24] all conclude inadequate quality of web-based information about COVID-19, confirming our findings using the same instruments. We also analyzed comprehensiveness with an inductive approach and found a substantial variability in regard to coverage of topics about both preventive measures and self care methods, with many topics being covered by very few websites. Our findings confirm the study from Hernández-García and Giménez-Júlvez, which showed variability in regard to comprehensiveness [25]. For example, few of the included websites in this study provided details on how to wash and sanitize hands even though these are very common and important preventive measures. Additionally, few of the included websites provided details concerning isolation when suspected or confirmed infection as well as how and when to contact health care services. These findings call attention to the risk of failing to inform members of the public about the specifics needed to ensure that preventive measures are not only implemented in their daily lives but also implemented correctly based on trustworthy recommendations. Taken together, the systematic instruments used in this study all illustrate low quality and confirm the conclusions of previous studies. Future studies should explore informational preferences in the general population and identify barriers to effective dissemination of high-quality information about COVID-19. Such studies need to take into account the wide range of preferences and barriers related to the multidimensional nature of the concept quality of health-related information [18], including but not limited to the quality criteria investigated in this study.

Judging from the findings of this and a numerous studies investigating the quality of web-based information about other health-related topics [12, 15, 16], there is an urgent need to identify innovative strategies and interventions that ensure the dissemination of high-quality web-based information and combats online misinformation. Health professionals who consult patients play an undisputed important role as advocates for healthy and nuanced behaviors related to the retrieval of web-based information. However, research has revealed various potential barriers of effective clinical communication, implicating a need for future interventions that aim to aid professionals in this endeavor [47]. Additional suggested strategies include engaging students and health professionals in the production of information to higher degrees [48] and utilizing methods that involve the public to co-design interventions capitalizing on the strengths of online communication [49]. Judging by our findings, we now urge developers, stakeholders and decision-makers to take novel actions to ensure higher quality of web-based information about COVID-19 as well as other current and future pandemics.

There are some methodological considerations that need to be considered when interpreting the findings of this study. We designed the searches with the intention to imitate search patterns in the general public. However, it is probable that we were not able to cover all various ways lay persons may search for web-based information. We used Google as the single search engine because it is currently the most popular search engine in Sweden (approximately 97% of the Swedish population use it to search for web-based information) [28]. It is possible that other search engines would have produced other hit lists leading to other websites than those included. On the other hand, we encountered 132 (39%) duplicate hits when screening for inclusion with various search strings, indicating that the included websites to a large extent represent the available Swedish web-based content at the time of the searches. We assessed quality through means of inductive analyses as well as systematic evaluations with three standardized instruments. Combined, these methods capture at least five different criteria of quality. We acknowledge that website quality encompasses a wide range of criteria that were not evaluated in this study, such as readability, accuracy and interactivity [50]. We encourage more research investigating other quality variables of web-based sources about COVID-19. A researcher with formal education and clinical experience in health care performed the assessments. To explore potential bias, another assessor, albeit also a researcher in health care, rated a subsample and the interrater reliability was excellent. Nevertheless, it is possible that other assessors such as lay persons in the general public would rate the websites differently. We encourage future studies using lay persons as assessors. Information about preventive measures and self care methods related to COVID-19 concerns all members of the public, regardless of socio-demographic background, and some populations such as older individuals and immigrants may have specific needs. Future research should take this into account and explore information specifically developed for subgroups in the population further. Moreover, the study context was Swedish websites about COVID-19, which may limit the generalizability. Worldwide, countries differs in regard to public interventions implemented with the purpose to reduce spread of the infection [4] and national methods for dissemination of information about the pandemic may also differ. We argue that our findings can be generalized to similar contexts but needs to be interpreted with caution and together with other studies when considered in countries that differ from the Swedish context.

## Conclusion

At the beginning of epidemics and pandemics, dissemination of high-quality information about preventive measures and self care methods is crucial in order to inform the public how to reduce the spread of the infection and manage symptoms at home. Judging from the findings of this study, it is probable that substantial quality deficits of websites about COVID-19 counteracted the public recommendations for preventive measures when the general population sought information during a new and unfamiliar situation. This illustrates a critical need for standardized and systematic routines on how to achieve dissemination of high-quality web-based information when new epidemics and pandemics emerge. There is a need to identify innovative strategies and interventions that ensure the dissemination of high-quality web-based information and combats online misinformation. Future studies should explore how members of the general public experience web-based information about these topics, investigate what type of sources they choose to rely on for this information, and identify barriers for successful dissemination of trustworthy high-quality information about preventive measures and self care methods of COVID-19.

## Additional Files

**Additional File 1.** Search strings and included hits.

**Additional File 2.** Instruments used for quality assessment.

**Additional File 3 **Medians, interquartile ranges and ranges of the investigated quality variables for the included websites (*n* = 76).

## Data Availability

The dataset used and analyzed during the current study is available from the corresponding author upon reasonable request.

## Abbreviations

COVID-19: Coronavirus disease 2019
JAMA: Journal of the American Medical Association
QUEST: The QUality Evaluation Scoring Tool

## References

[1] Xie Y, Wang Z, Liao H, Marley G, Wu D, Tang W (2020). Epidemiologic, clinical, and laboratory findings of the COVID-19 in the current pandemic: systematic review and meta-analysis. BMC Infect Dis.

[2] Arefi MF, Poursadeqiyan M (2020). A review of studies on the COVID-19 epidemic crisis disease with a preventive approach. Work..

[3] Rios P, Radhakrishnan A, Williams C, Ramkissoon N, Pham B, Cormack GV (2020). Preventing the transmission of COVID-19 and other coronaviruses in older adults aged 60 years and above living in long-term care: a rapid review. Syst Rev.

[4] Patiño-Lugo DF, Vélez M, Velásquez Salazar P, Vera-Giraldo CY, Vélez V, Marín IC (2020). Non-pharmaceutical interventions for containment, mitigation and suppression of COVID-19 infection. Colomb Med (Cali).

[5] The Royal Australian College of General Practitioners. Home-care guidelines for adult patients with mild COVID-19. 2020. https://www.racgp.org.au/FSDEDEV/media/documents/Clinical%20Resources/Guidelines/Home-care-guidelines-for-adult-patients-with-mild-COVID-19.pdf.

[6] National Institute for Health and Care Excellence. COVID-19 rapid guideline: managing symptoms (including at the end of life) in the community. 2020. https://www.nice.org.uk/guidance/ng163/resources/covid19-rapid-guideline-managing-symptoms-including-at-the-end-of-life-in-the-community-pdf-66141899069893. 33497151

[7] The National Board of Health and Welfare. Triage/flöden och arbetssätt vid covid-19 [Triage/processes and work during covid-19]. 2020. https://www.socialstyrelsen.se/globalassets/sharepoint-dokument/dokument-webb/ovrigt/triage-arbetssatt-vardcentral-covid19.pdf.

[8] Anwar A, Malik M, Raees V, Anwar A (2020). Role of mass media and public health communications in the COVID-19 pandemic. Cureus..

[9] Le HT, Nguyen DN, Beydoun AS, Le XTT, Nguyen TT, Pham QT, et al. Demand for health information on COVID-19 among Vietnamese. Int J Environ Res Public Health. 2020;17(12). 10.3390/ijerph17124377. 10.3390/ijerph17124377 PMC7344690 32570819

[10] Jo W, Lee J, Park J, Kim Y (2020). Online information exchange and anxiety spread in the early stage of the novel coronavirus (COVID-19) outbreak in South Korea: structural topic model and network analysis. J Med Internet Res.

[11] Cline RJ, Haynes KM (2001). Consumer health information seeking on the internet: the state of the art. Health Educ Res.

[12] Eysenbach G, Powell J, Kuss O, Sa E-R (2002). Empirical studies assessing the quality of health information for consumers on the world wide web: a systematic review. JAMA..

[13] Venot A, Burgun A, Quantin C (2014). Medical informatics, e-health: fundamentals and applications.

[14] Abdel-Wahab N, Rai D, Siddhanamatha H, Dodeja A, Suarez-Almazor ME, Lopez-Olivo MA (2019). A comprehensive scoping review to identify standards for the development of health information resources on the internet. PLoS One.

[15] Daraz L, Morrow AS, Ponce OJ, Beuschel B, Farah MH, Katabi A, Alsawas M, Majzoub AM, Benkhadra R, Seisa MO, Ding J(F), Prokop L, Murad MH (2019). Can patients trust online health information? A meta-narrative systematic review addressing the quality of health information on the internet. J Gen Intern Med.

[16] Daraz L, Morrow AS, Ponce OJ, Farah W, Katabi A, Majzoub A, Seisa MO, Benkhadra R, Alsawas M, Larry P, Murad MH (2018). Readability of online health information: a meta-narrative systematic review. Am J Med Qual.

[17] Eysenbach G (2009). Infodemiology and infoveillance: framework for an emerging set of public health informatics methods to analyze search, communication and publication behavior on the internet. J Med Internet Res.

[18] Sun Y, Zhang Y, Gwizdka J, Trace CB (2019). Consumer evaluation of the quality of online health information: systematic literature review of relevant criteria and indicators. J Med Internet Res.

[19] Niu L, Luo D, Liu Y, Xiao S. The accessibility, usability, and reliability of Chinese web-based information on HIV/AIDS. Int J Environ Res Public Health. 2016;13(8). 10.3390/ijerph13080834. 10.3390/ijerph13080834 PMC4997520 27556475

[20] Bora K, Das D, Barman B, Borah P (2018). Are internet videos useful sources of information during global public health emergencies? A case study of YouTube videos during the 2015-16 Zika virus pandemic. Pathog Glob Health.

[21] Fan KS, Ghani SA, Machairas N, Lenti L, Fan KH, Richardson D, Scott A, Raptis DA (2020). COVID-19 prevention and treatment information on the internet: a systematic analysis and quality assessment. BMJ Open.

[22] Joshi A, Kajal F, Bhuyan SS, Sharma P, Bhatt A, Kumar K (2020). Quality of novel coronavirus related health information over the internet: an evaluation study. ScientificWorldJournal..

[23] Jayasinghe R, Ranasinghe S, Jayarajah U, Seneviratne S (2020). Quality of online information for the general public on COVID-19. Patient Educ Couns.

[24] Cuan-Baltazar JY, Muñoz-Perez MJ, Robledo-Vega C, Pérez-Zepeda MF, Soto-Vega E (2020). Misinformation of COVID-19 on the internet: Infodemiology study. JMIR Public Health Surveill.

[25] Hernández-García I, Giménez-Júlvez T (2020). Assessment of health information about COVID-19 prevention on the internet: Infodemiological study. JMIR Public Health Surveill.

[26] Rew L, Saenz A, Walker LO (2018). A systematic method for reviewing and analysing health information on consumer-oriented websites. J Adv Nurs.

[27] The Public Health Agency of Sweden. Our mission. 2018. http://www.folkhalsomyndigheten.se/the-public-health-agency-of-sweden/about-us/our-mission/. Accessed 13 Nov 2020.

[28] The Internet Foundation In Sweden. Svenskarna och internet 2018 [Swedes and the internet 2018]. 2018. https://www.iis.se/docs/Svenskarna_och_internet_2018.pdf. Accessed 28 Dec 2018.

[29] Eysenbach G, Köhler C (2002). How do consumers search for and appraise health information on the world wide web? Qualitative study using focus groups, usability tests, and in-depth interviews. BMJ..

[30] Peterson G, Aslani P, Williams KA (2003). How do consumers search for and appraise information on medicines on the internet? A qualitative study using focus groups. J Med Internet Res.

[31] Fiksdal AS, Kumbamu A, Jadhav AS, Cocos C, Nelsen LA, Pathak J, McCormick JB (2014). Evaluating the process of online health information searching: a qualitative approach to exploring consumer perspectives. J Med Internet Res.

[32] Feufel MA, Stahl SF (2012). What do web-use skill differences imply for online health information searches?. J Med Internet Res.

[33] Graneheim UH, Lundman B (2004). Qualitative content analysis in nursing research: concepts, procedures and measures to achieve trustworthiness. Nurse Educ Today.

[34] Silberg WM, Lundberg GD, Musacchio RA (1997). Assessing, controlling, and assuring the quality of medical information on the internet: Caveant lector et viewor--let the reader and viewer beware. JAMA..

[35] Robillard JM, Jun JH, Lai J-A, Feng TL (2018). The QUEST for quality online health information: validation of a short quantitative tool. BMC Med Inform Decis Mak.

[36] McCool ME, Wahl J, Schlecht I, Apfelbacher C (2015). Evaluating written patient information for eczema in German: comparing the reliability of two instruments, DISCERN and EQIP. PLoS ONE.

[37] Charnock D, Shepperd S, Needham G, Gann R (1999). DISCERN: an instrument for judging the quality of written consumer health information on treatment choices. J Epidemiol Community Health.

[38] Charnock D. The DISCERN handbook: quality criteria for consumer health information on treatment choices. Discern online 1998. http://www.discern.org.uk/discern.pdf. Accessed 2 Jan 2016.

[39] Griffiths KM, Christensen H (2005). Website quality indicators for consumers. J Med Internet Res.

[40] Cicchetti DV (1994). Guidelines, criteria, and rules of thumb for evaluating normed and standardized assessment instruments in psychology. Psychol Assess.

[41] Helsingen LM, Refsum E, Gjøstein DK, Løberg M, Bretthauer M, Kalager M (2020). The COVID-19 pandemic in Norway and Sweden - threats, trust, and impact on daily life: a comparative survey. BMC Public Health.

[42] Rovetta A, Bhagavathula AS (2020). COVID-19-related web search behaviors and Infodemic attitudes in Italy: Infodemiological study. JMIR Public Health Surveill.

[43] Kummervold PE, Wynn R (2012). Health information accessed on the internet: the development in 5 European countries. Int J Telemed Appl.

[44] Wynn R, Oyeyemi SO, Budrionis A, Marco-Ruiz L, Yigzaw KY, Bellika JG (2020). Electronic health use in a representative sample of 18,497 respondents in Norway (the seventh Tromsø study - part 1): population-based questionnaire study. JMIR Med Inform.

[45] van Dijk JAGM (2006). Digital divide research, achievements and shortcomings. Poetics..

[46] Okan O, Bollweg TM, Berens E-M, Hurrelmann K, Bauer U, Schaeffer D. Coronavirus-related health literacy: a cross-sectional study in adults during the COVID-19 Infodemic in Germany. Int J Environ Res Public Health. 2020;17(15). 10.3390/ijerph17155503. 10.3390/ijerph17155503 PMC7432052 32751484

[47] Tan SS-L, Goonawardene N (2017). Internet health information seeking and the patient-physician relationship: a systematic review. J Med Internet Res.

[48] Azzam A, Bresler D, Leon A, Maggio L, Whitaker E, Heilman J, Orlowitz J, Swisher V, Rasberry L, Otoide K, Trotter F, Ross W, McCue JD (2017). Why medical schools should embrace Wikipedia: final-year medical student contributions to Wikipedia articles for academic credit at one school. Acad Med.

[49] Richards T, Scowcroft H (2020). BMJ’s international patient and public advisory panel. Patient and public involvement in covid-19 policy making. BMJ.

[50] Burkell J (2004). Health information seals of approval: what do they signify?. Inf Commun Soc.

