# Supplementary material for: Quality of web-based information at the beginning of a global pandemic: a cross-sectional infodemiology study investigating preventive measures and self care methods of the coronavirus disease 2019
Source: BMC Public Health. 2021 Jun 14;21:1141. doi: 10.1186/s12889-021-11141-9 (PMC8201436; doi:10.1186/s12889-021-11141-9)
Supplement: Supplementary file 1 — Additional File 1. Search strings and included hits. [file 12889_2021_11141_MOESM2_ESM.pdf]

Additional File 2. Instruments used for quality assessment.

Instrument [Quality criteria]	Subscale, benchmark or indicator	Questions or aspects	Score
Content analysis [Comprehensiveness]	Inductive analysis	Subcategories concerning preventive measures	0-33
		Subcategories concerning self care methods	0-30
		Total number of subcategories	0-63
JAMA [Transparency]	Authorship	Names, affiliations and relevant credentials of authors and contributors provided	0-1
	Attribution	References and sources for all content are listed clearly, and all relevant copyright information is noted	0-1
	Disclosure	Website ownership, sponsorship, advertising, underwriting, commercial funding/support and conflicts of interest prominently and fully disclosed	0-1
	Currency	Dates when content was posted and updated are indicated	0-1
	Total score		0-4
DISCERN [Reliability and quality of information]	Reliability	Are the aims clear?	1-5
		Does it achieve its aims? (only answered if 2-5 in first question)	1-5
		Is it relevant?	1-5
		Is it clear what sources of information were used to compile the publication (other than the author or producer)?	1-5
		Is it clear when the information used or reported in the publication was produced?	1-5
		Is it balanced and unbiased?	1-5
		Does it provide details of additional sources of support and information?	1-5
		Does it refer to areas of uncertainty?	1-5
		Total score subscale 1 (Reliability)	7-40
	Quality of information[1]	Does it describe how each *preventive measure and/or self care method* works?	1-5
		Does it describe the benefits of each *preventive measure and/or self care* method?	1-5
		Does it describe the risks of each *preventive measure and/or self care* method?	1-5
		Does it describe what would happen if no *preventive measure and/or self care* method is used?	1-5
		Does it describe how *preventive measures and/or self care* methods affect overall quality of life?	1-5
		Is it clear that there may be more than one *possible preventive measure and/or self care* method?	1-5
		Does it provide support for shared decision-making *and prompts when to contact heath care*?	1-5
		Total score subscale 2 (Quality of information)	7-35
	Overall rating	Based on the answers to all of the above questions, rate the overall quality of the publication as a source of information	1-5
	Total DISCERN score		15-80
QUEST [Quality of online sources about disease prevention]	Authorship	0: No indication of authorship or username<br>1: All other indications of authorship<br>2: Author's name and qualification clearly stated	0-2
	Attribution	0: No sources<br>1: Mention of expert source, research findings (though with insufficient information to identify the specific studies), links to various sites, advocacy body, or other<br>2: Reference to at least one identifiable scientific study, regardless of format (e.g., information in text, reference list)<br>3: Reference to mainly identifiable scientific studies, regardless of format (in >50% of claims)	0-9[2]
	Type of study (Only when attribution 2/3)	0: In vitro, animal models, or editorials<br>1: All observational work<br>2: Meta-analyses, randomized controlled trials, clinical studies	0-2

Conflict of interest	0: Endorsement or promotion of intervention designed to prevent or treat condition (e.g., supplements, brain training games, foods) within the article<br>1: Endorsement or promotion of educational products & services (e.g., books, care home services)<br>2: Unbiased information	0-6[2]
Currency	0: No date present<br>1: Article is dated but 5 years or older<br>2: Article is dated within the last 5 years	0-2
Complementarity	0: No support of the patient-health professional relationship<br>1: Support of the patient-health professional relationship	0-1
Tone	0: Fully supported (authors fully and unequivocally support the claims, strong vocabulary such as “cure”, “guarantee”, and “easy”, mostly use of non-conditional verb tenses (“can”, “will”), no discussion of limitations)<br>1: Mainly supported (authors mainly support their claims but with more cautious vocabulary such as “can reduce your risk” or “may help prevent”, no discussion of limitations)<br>2: Balanced/cautious support (authors’ claims are balanced by caution, includes statements of limitations and/or contrasting findings)	0-6[2]
Total QUEST score		0-28

[1]Questions modified so that the questions concerned preventive measures and self care methods (changed part in questions in italics)
[2]Weighted score
