# Supplementary material for: Quality of web-based information at the beginning of a global pandemic: a cross-sectional infodemiology study investigating preventive measures and self care methods of the coronavirus disease 2019
Source: BMC Public Health. 2021 Jun 14;21:1141. doi: 10.1186/s12889-021-11141-9 (PMC8201436; doi:10.1186/s12889-021-11141-9)
Supplement: Supplementary file 2 — Additional File 2. Instruments used for quality assessment. [file 12889_2021_11141_MOESM3_ESM.pdf]

Additional File 3. Medians, interquartile ranges and ranges of the investigated quality variables for the included websites (n=76) [minimum to maximum achievable score in square brackets].

	Government		Health care		Newspapers		Information websites		Pharmacy		Other		Total sample	
**Quality criteria (instrument)**	**Md (IQR)**	**Range**	**Md (IQR)**	**Range**	**Md (IQR)**	**Range**	**Md (IQR)**	**Range**	**Md (IQR)**	**Range**	**Md (IQR)**	**Range**	**Md (IQR)**	**Range**
Comprehensiveness (content analysis)														
Included subcategories about preventive measures [0-33]	9.0 (5.0)	2-14	5.0 (5.0)	0-13	4.0[2] (4.0)	0-12	7.0 (4.0)	2-16	6.0 (2.0)	4-9	7.0 (4.0)	3-13	6.0 (5.0)	0-16
Included subcategories about self care [0-30]	3.0 (3.0)	0-17	4.0 (3.0)	0-15	2.0 (3.0)	0-7	4.0 (3.0)	0-8	5.0 (0)	0-5	1.0 (2.0)	0-9	3.0 (3.25)	0-17
Total subcategories [0-63]	12.0 (6.5)	2-20	9.0 (6.0)	2-26	6.0 (7.0)	1-16	13.0 (10.0)	2-21	11.0 (3.0)	5-14	9.0 (5.0)	3-16	10.0 (8.0)	1-26
Transparency (JAMA benchmarks)														
Number of benchmarks achieved	0[3,4] (0.5)	0-2	0[3,4] (1.0)	0-1	1.0 (1.0)	0-3	3.0 (2.0)	0-4	0[3,4] (0)	0-0	0[3] (1.0)	0-1	0 (1.0)	0-4
Quality of online sources about disease prevention (QUEST)														
Authorship [0-2]	0[3,4,5] (0)	0-0	0 (2.0)	0-2	1.0 (1.0)	0-2	2.0 (0)	0-2	0[3] (0)	0-0	0[3] (0)	0-1	0 (1.0)	0-2
Attribution [0-9]	0[3,4] (3.0)	0-3	0[3,4] (3.0)	0-3	3.0 (0)	0-6	3.0 (0)	3-9	3.0 (0)	0-3	3.0 (0)	0-3	3.0 (3.0)	0-9
Attribution 2[1] [0-2]	-	-	-	-	1 (-)	1-1	1 (-)	1-1	-	-	-	-	1 (-)	1
Conflict of interest [0-6]	6.0 (0)	6-6	6.0[2] (3.0)	3-6	6.0 (0)	3-6	6.0 (0)	0-6	6.0 (6.0)	0-6	6.0 (0)	0-6	6.0 (0)	0-6
Currency [0-2]	2.0 (1.0)	0-2	2 (2.0)	0-2	2.0 (0)	0-2	2.0 (0)	0-2	0[4] (0)	0-2	2.0 (2.0)	0-2	2.0 (2.0)	0-2
Complementarity [0-1]	1.0 (0)	0-1	1.0 (0)	0-1	1.0 (1)	0-1	1.0 (0)	0-1	1.0 (0)	1-1	0[5] (1.0)	0-1	1.0 (1.0)	0-1
Tone [0-6]	3.0 (0)	0-6	3.0 (0)	0-3	3.0 (3.0)	0-6	3.0 (3.0)	0-6	3.0 (0)	0-3	3.0 (0)	3-3	3.0 (0)	0-6
Total score [0-28]	12.0[3] (4.5)	8-18	12.0[3] (7.0)	7-17	14.0 (4.0)	10-23	16.0 (3.0)	13-19	9.0[3] (6.0)	7-13	14.0 (2.0)	6-15	13.0 (3.25)	6-23
Reliability and quality of consumer health information (DISCERN)														
Subscale 1 (reliability) [7-40]	15.0 (5.0)	9-23	14.0 6.0)	10-20	14.0 (4.0)	10-30	15.0 (9.0)	11-26	13.0 (2.0)	10-16	13.0 (4.0)	10-28	14.5 (5.0)	9-30
Subscale 2 (information about prevention and self care) [7-35]	13.0 (3.5)	7-20	13.0 (5.0)	8-18	9.0 (5.0)	7-19	13.0 (6.0)	8-21	12.0 (1.0)	10-16	12.0 (6.0)	9-17	12.0 (5.0)	7-21
Subscale 3 (overall quality) [1-5]	2.0 (1.0)	1-4	2.0 (0)	1-4	1.0 (1.0)	1-4	3.0 (1.0)	1-4	2.0 (0)	1-3	2.0 (1.0)	2-3	2.0 (1.0)	1-4
Total score [15-80]	30.0 (8.0)	19-47	29.0 (6.0)	21-42	24.0 (10.0)	19-53	33.0 (15.0)	21-51	27.0 (4.0)	24-32	28.0 (6.0)	22-47	29.0 (9.0)	19-53

[1]Follow-up attribution score only applicable for two websites
[2]Significantly lower than government affiliation
[3]Significantly lower than information website affiliation
[4]Significantly lower than newspaper affiliation
[5]Significantly lower than health care affiliation
