# Supplementary material for: Quality of web-based information at the beginning of a global pandemic: a cross-sectional infodemiology study investigating preventive measures and self care methods of the coronavirus disease 2019
Source: BMC Public Health. 2021 Jun 14;21:1141. doi: 10.1186/s12889-021-11141-9 (PMC8201436; doi:10.1186/s12889-021-11141-9)
Supplement: Supplementary file 3 — Additional File 3 Medians, interquartile ranges and ranges of the investigated quality variables for the included websites (n = 76). [file 12889_2021_11141_MOESM1_ESM.pdf]

Additional File 1. Search strings and included hits.

Search string in Swedish	Search string translated in English	Total hits	Included hits	
			Unique	Duplicate
Coronavirus förebygga	*Coronavirus prevention*	1,070,000	12	0
Covid förebygga	*Covid prevention*	461,000	1	4
Covid-19	*Covid-19*	4,050,000,000	3	1
Hur undviker jag corona	*How do I avoid corona*	4,200,000	10	8
Hur undviker jag covid-1913	*How do I avoid covid-19*	6,000,000	7	10
Hur förhindrar jag att smittas av corona	*How do I prevent being infected by corona*	1,160,000	5	11
Hur förhindrar jag att smittas av covid-19	*How do I prevent being infected by covid-19*	1,290,000	1	14
Hur skyddar jag mig från corona	*How do I protect myself from corona*	1,470,000	6	13
Hur skyddar jag mig från covid-19	*How do I protect myself from covid-19*	4,400,000	3	16
Egenvård corona	*Self care corona*	27,400	8	4
Egenvård covid-19	*Self care covid-19*	66,800	2	11
Hur lindrar jag coronainfektion	*How do I alleviate corona infection*	219,000	8	7
Hur lindrar jag covid-19	*How do I alleviate covid-19*	371,000	2	11
SARS-CoV-2	*SARS-CoV-2*	470,000,000	1	1
Virus corona	*Virus corona*	882,000,000	3	6
Coronavirus tips	*Coronavirus tips*	9,930,000,000	3	4
Coronavirus symtom	Coronavirus symptoms	1,150,000	1	11
**Total for all search strings**			**76**	**132**
